# Quantitative Trait Loci Mapping for Bacterial Blight Resistance in Rice Using Bulked Segregant Analysis

**DOI:** 10.3390/ijms150711847

**Published:** 2014-07-03

**Authors:** Xueying Han, Yong Yang, Xuming Wang, Jie Zhou, Wenhao Zhang, Chulang Yu, Chen Cheng, Ye Cheng, Chengqi Yan, Jianping Chen

**Affiliations:** 1 College of Plant Protection, Nanjing Agricultural University, Nanjing 210095, China; E-Mail: 2011102004@njau.edu.cn; 2 State Key Laboratory Breeding Base for Zhejiang Sustainable Pest and Disease Control, Ministry of Agriculture Key Laboratory of Biotechnology in Plant Protection, Zhejiang Provincial Key Laboratory of Plant Virology, Institute of Virology and Biotechnology, Zhejiang Academy of Agricultural Science, Hangzhou 310021, China; E-Mails: zaasyangyong@gmail.com (Y.Y.); wangxuming@gmail.com (X.W.); zaaszhoujie@gmail.com (J.Z.); yuchulang@163.com (C.Y.); anson_cheng@126.com (C.C.); chengye1969@163.com (Y.C.); 3 College of Life Sciences, China Jiliang University, Hangzhou 310018, China; E-Mail: zwh18767163155@126.com

**Keywords:** quantitative trait loci, mapping, bacterial blight resistance, bulked segregant analysis, *Oryza meyeriana*

## Abstract

*Oryza meyeriana* is highly resistant to rice bacterial blight (BB) and this resistance trait has been transferred to cultivated rice (*O. sativa*) using asymmetric somatic hybridization. However, no resistance genes have yet been cloned. In the present study, a progeny of the somatic hybridization with high BB resistance was crossed with a rice cultivar with high BB susceptibility to develop an F_2_ population. Using bulked segregant analysis (BSA), 17 polymorphic markers that were linked to rice BB resistance were obtained through scanning a total of 186 simple sequence repeats (SSR) and sequence-tagged site (STS) markers, evenly distributed on 12 chromosomes. A genetic linkage map was then constructed based on the 17 linkage markers and the F_2_ segregating population, which was followed by mapping for quantitative trait loci (QTLs) for BB resistance. Three QTLs were identified on chromosomes 1, 3 and 5, respectively, and the alleles of the resistant parent at any of the QTLs increased BB resistance. All of the three QTLs had a strong effect on resistance, explaining about 21.5%, 12.3% and 39.2% of the resistance variance, respectively. These QTLs were different from the loci of the BB resistance genes that have been identified in previous studies. The QTLs mapped in this work will facilitate the isolation of novel BB resistance genes and their utilization in rice resistance breeding.

## 1. Introduction

*Xanthomonas oryzae* pv. *oryzae* (*Xoo*) causes rice bacterial blight (BB), one of the most destructive bacterial diseases of rice worldwide. It is responsible for severe yield losses to global rice production, generally in the range of 20% to 30%, and as high as 50% in years when the disease is prevalent [1]. Chemical control is temporarily effective but often causes environmental pollution. In contrast, methods that depend on the plant’s innate immune system are more acceptable and environmentally friendly. So far, at least 36 genes conferring resistance to various *Xoo* strains have been identified [1,2,3,4]. Most of them have been tagged with DNA markers and eight of them (*Xa1*, *Xa3/Xa26*, *Xa5*, *Xa10*, *Xa13*, *Xa21*, *Xa25*, *Xa27*) have been cloned successfully using a map-based cloning strategy [3,4,5,6,7,8,9,10]. Some of these resistance genes have been widely used in rice breeding for BB resistance and many useful cultivars have been released in Asian countries.

However, development of resistant cultivars always faces difficulties in terms of durability of resistance because of the diversity and variability of the *Xoo* pathogen. For example, the resistance gene *Xa4* has been deployed in many resistant varieties that have played an important role in defense against rice BB disease in the tropics and in many parts of China [11,12]. However, many cultivars only carrying *Xa4* have now become sensitive to the disease because of the spread of *Xoo* strains able to overcome the resistance [12]. *Xa21* has been widely used as a broad spectrum resistance gene since it was identified in the 1990s, but new pathogen virulence that can overcome *Xa21* has also been found in many areas of Korea, Philippines, Japan and China [13,14]. Therefore, it is always important for researchers to exploit new resistance genes and this strategy plays an important role in durable BB resistance of rice.

*Oryza meyeriana* is one of the most important wild rice resources identified in South and Southeast Asia. It is adapted to survive in harsh environments and possesses many useful traits absent in cultivated rice, including high resistance to BB [15,16,17,18]. However, no resistance genes have yet been cloned in *O. meyeriana*, which has delayed the utilization of this material in rice breeding. One important reason is that conventional crossing methods fail to generate fertile hybrids between cultivated rice and *O. meyeriana* because the two species contain different types of genome (AA and GG respectively) [19]. Somatic hybridization through the fusion of two somatic protoplasts has already been used to overcome sexual incompatibility and produce inter-species hybrids, and the improved technique of asymmetric somatic hybridization has been used to reduce the co-introduction of deleterious traits from the donor species [20]. Using this breeding technique, we have introduced BB resistance genes from *O. meyeriana* to a BB susceptible rice cultivar Dalixiang [17]. ASH1 (Asymmetric Somatic Hybridization 1) is one of the hybrid progenies combining the high BB resistance of *O. meyeriana* with the good agronomic characters of Dalixiang, and is an ideal intermediate material for cloning the novel resistance gene(s) of *O. meyeriana*. Our long-term aim is therefore to clone the resistance gene(s) in ASH1 through a map-based cloning strategy. In this work, we mapped quantitative trait loci (QTLs) for BB resistance in ASH1, using bulked segregant analysis (BSA), in order to provide a basis for further cloning of the resistance gene(s) and their utilization in rice breeding.

## 2. Results

### 2.1. Resistance Analysis

**Figure 1:**
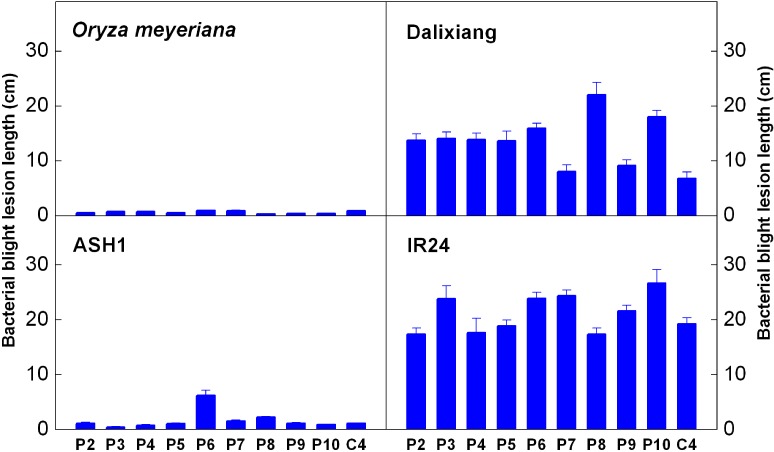
The resistance to rice bacterial blight (BB) of *Oryza meyeriana*, Dalixiang, ASH1 and IR24. Rice was inoculated by the leaf-clipping method with ten different *Xanthomonas oryzae* pv. *oryzae* (*Xoo*) strains, and for each strain, at least 16 leaves from four plants were inoculated. Lesion length was measured three weeks after inoculation. Bars indicate the standard error.

**Figure 2:**
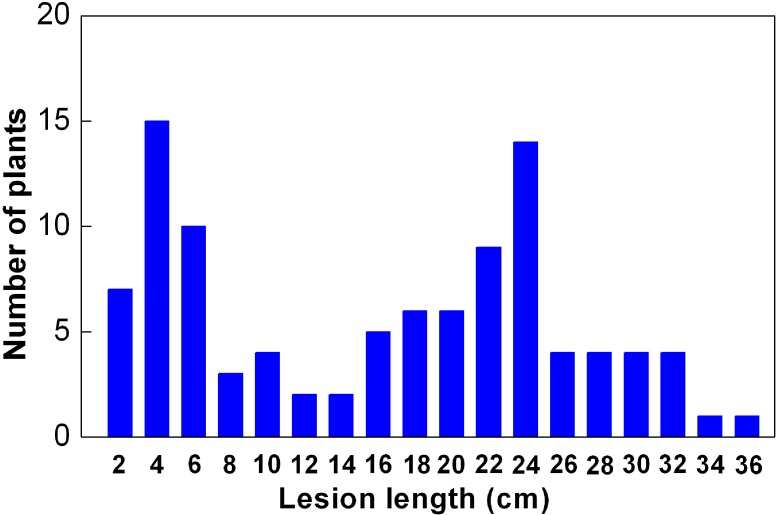
Frequency distribution of bacterial blight lesion length in the F_2_ population derived from ASH1/IR24. Rice was inoculated by the leaf-clipping method with *Xoo* strain P10, and at least eight leaves were inoculated for each plant. Lesion length was measured three weeks after inoculation.

*O. meyeriana*, one parent of the asymmetric somatic hybridization, was highly resistant to all *Xoo* strains while the other parent, cultivated rice Dalixiang, was highly susceptible to almost all of the strains (Figure 1). The hybrid progeny ASH1 shared the high BB resistance of the wild rice (Figure 1). Because ASH1 was derived from the *japonica* cultivar Dalixiang, an *indica* cultivar IR24 was selected as another parent for QTL mapping in order to produce marker polymorphism between the parents. It has been widely reported that IR24 is highly susceptible to BB disease, which was confirmed by the data in this study (Figure 1). The significant difference in BB resistance between ASH1 and IR24 was favorable to resistance segregation in the F_2_ population. The frequency distribution in the F_2_ population exhibited a bimodal pattern with the values ranging from 1.08 to 34.43 cm (Figure 2).

### 2.2. Markers Linked to Bacterial Blight (BB) Resistance

**Figure 3:**
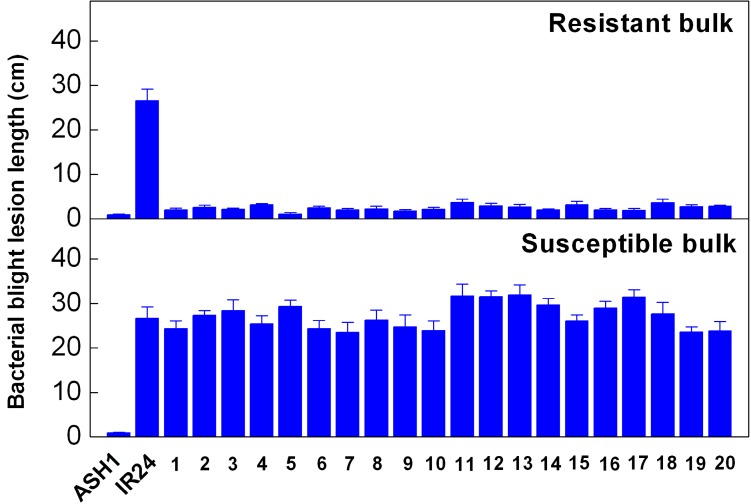
Resistance of the rice plants in the resistant and susceptible bulk samples. Rice was inoculated by the leaf-clipping method with *Xoo* strain P10, and at least eight leaves were inoculated for each plant. Lesion length was measured three weeks after inoculation. The upper panel shows the lesion length of the 20 individuals from the resistant bulk, and the lower panel the susceptible bulk. Bars indicate the standard error.

Bulked segregant analysis (BSA) was performed according to Michelmore *et al.* [21] and Zhang *et al.* [22]. 20 resistant and 20 susceptible individuals were selected from the F_2_ population, respectively (Figure 3), and two DNA bulks, R (resistant) and S (susceptible), were made by mixing equal amounts of DNA from these selected plants.

**Figure 4:**
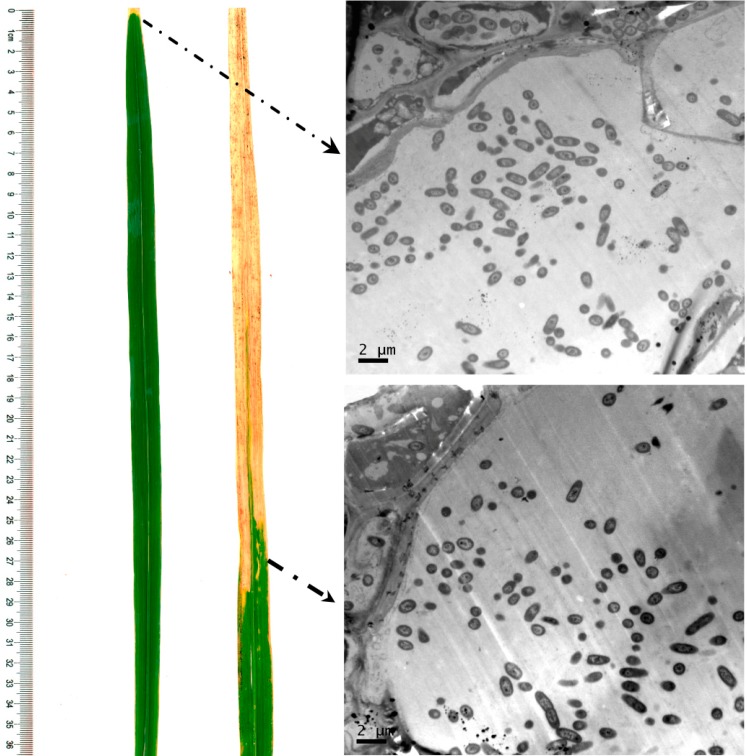
Electron micrograph showing the bacteria in xylem vessels in both extremely resistant and susceptible individuals from the two bulks. Left leaf and upper image were from an extremely resistant plant, and the right leaf and lower image from an extremely susceptible plant.

The success of the bacterial inoculation is important for resistance evaluation because a rice leaf without bacterial invasion has a similar phenotype to one with high resistance, and therefore a rice plant without successful inoculation could be mis-evaluated as a resistant individual. We therefore observed the bacterial invasion in rice leaves by electron microscopy to improve the resistance evaluation. The results showed that xylem vessels at the site near the visible lesion were filled with bacteria not only in the extremely susceptible plants but also in the extremely resistant ones (Figure 4), which confirmed the resistance identification of the individuals from the two bulks.

**Figure 5:**
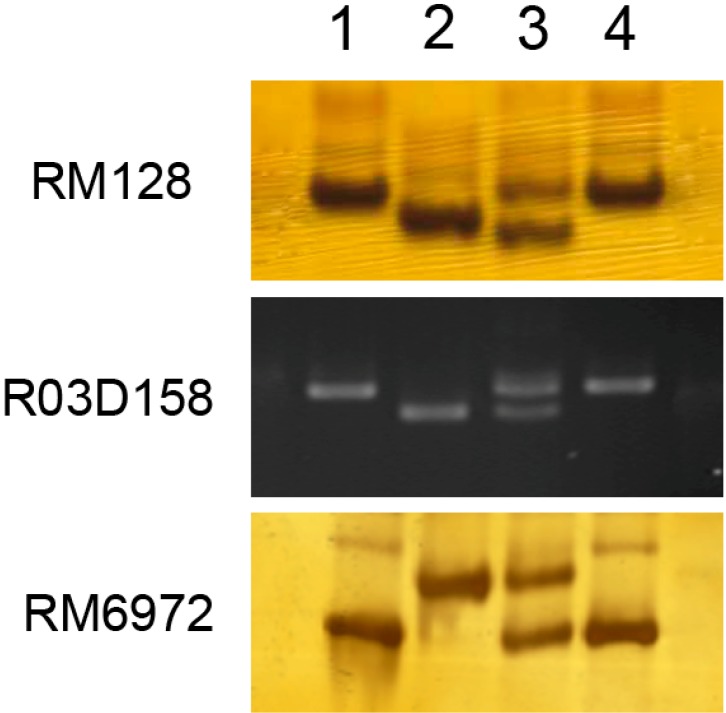
Banding patterns of three linkage markers RM128, R03D158 and RM6972 in two parents and two bulks. The numbers **1**, **2**, **3** and **4** represent ASH1, IR24, susceptible bulk and resistant bulk, respectively. RM128 is located on chromosome 1, R03D158 is located on chromosome 3, and RM6972 is located on chromosome 5.

A total of 698 molecular markers including simple sequence repeats (SSR) and sequence-tagged site (STS) markers were screened for polymorphism between the parents of ASH1 and IR24, and 186 markers evenly distributed on 12 rice chromosomes were selected due to their polymorphism and reproducible amplification (Table S1). Subsequently, these 186 markers were used to scan the two parents and the two bulks (R bulk and S bulk) and 17 markers were identified that showed linkage to BB resistance (Table S2). Of these 17 markers, 4 were on chromosome 1, 6 on chromosome 3 and 7 on chromosome 5 (Table S2). Figure 5 illustrates the band display patterns in two parents and two bulks of the three markers RM128 (chromosome 1), R03D158 (chromosome 3) and RM6972 (chromosome 5).

The linkage markers showed heterozygous patterns from both parents in the S bulk and uniformly homozygous patterns of the resistant parent in the R bulk (Figure 5). It is most likely that the identified loci correspond to recessive resistance genes. The recessive genes for BB resistance such as *Xa5* and *Xa13* have quantitative, additive and broad-spectrum resistance. ASH1 possesses BB resistance with broad-spectrum (Figure 1) and the resistance loci identified here are quantitative and additive. All the results are consistent with the character of recessive genes. However, this speculation needs to be examined by further detailed studies including the separation and resistance evaluation of different resistance loci and the cloning of the corresponding resistance genes.

### 2.3. Quantitative Trait Loci (QTL) Mapping

**Figure 6:**
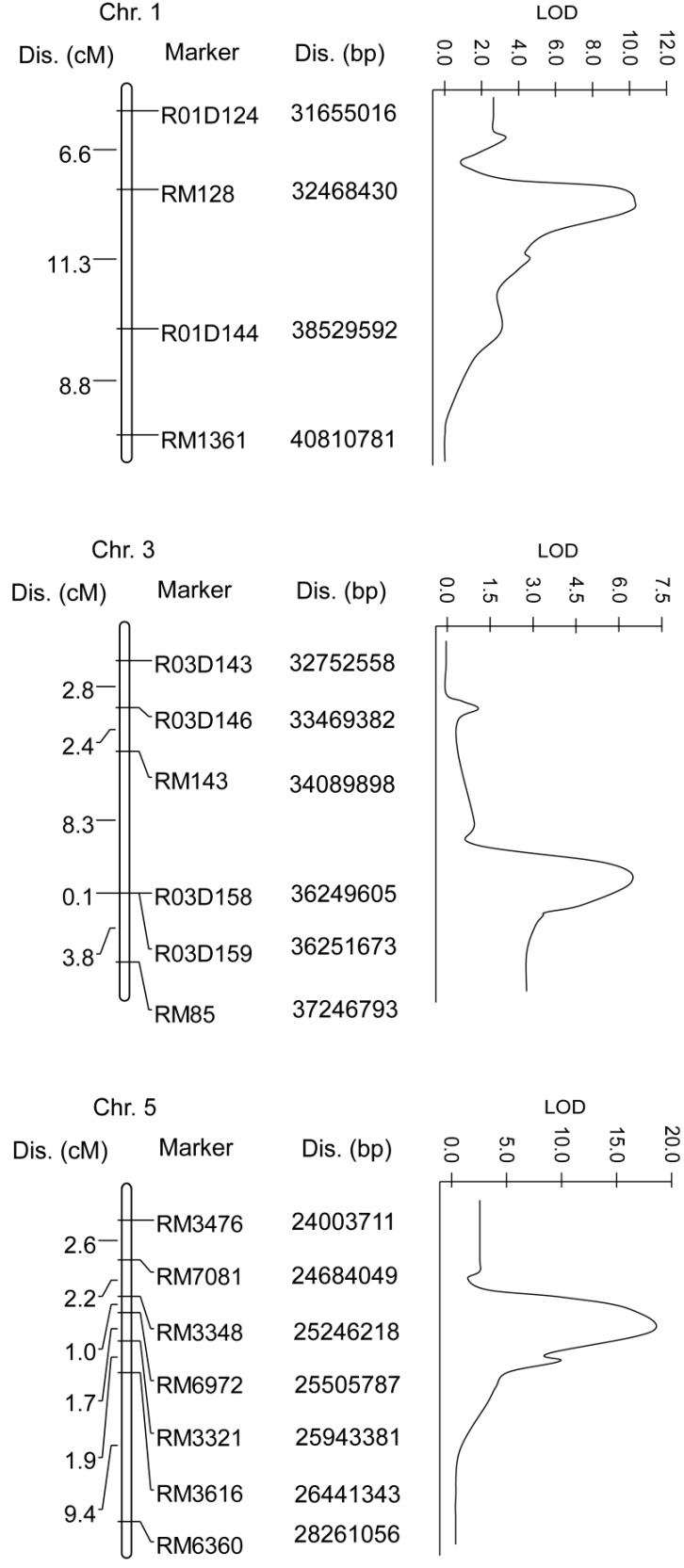
Mapping of the quantitative trait loci (QTLs) for BB resistance in the F_2_ population derived from ASH1/IR24. Upper, middle and bottom panels show the locations of qBBR1, qBBR3 and qBBR5, respectively. Genetic distance (cM) between adjacent markers and marker names are indicated on the left and right sides of the chromosomes. Physical distances (bp) of the markers on the chromosomes are also shown. The right part of each panel shows the graph for the distribution of the logarithm of odds (LOD) score.

To confirm the chromosome loci underlying BB resistance in ASH1, we further performed QTL analysis in the F_2_ population including 101 individuals using the obtained linkage markers. Three QTLs conferring BB resistance were detected on chromosomes 1, 3 and 5 (Figure 6, Table 1). ASH1 alleles at any of the three QTLs contributed to increased BB resistance. A QTL on chromosome 1, designated qBBR1 (QTL for BB resistance on chromosome 1), was located in the marker interval between RM128 and R01D144 with the nearest marker of RM128. qBBR1 explained about 21.5% of the phenotypic variance with an logarithm of odds (LOD) score of 10.1. qBBR3, with LOD score of 6.2, explained approximately 12.3% of the total resistance variance. This locus was targeted in the marker vicinity between R03D158 and RM85 on chromosome 3 with the nearest marker of R03D158. qBBR5 was mapped in the marker interval between RM7081 and RM3616 on chromosome 5, and the nearest marker was RM6972. The LOD score of qBBR5 was 17.8 and this QTL accounted for the highest resistance variance (about 39.2%). The LOD score of the three QTLs were all higher than 6.0 and they each explained more than 12% of resistance variance, which indicated that all of them were important QTLs conferring BB resistance.

**Table 1:** QTLs for rice BB resistance detected in ASH1.

QTL	Chromosome	Marker Interval	Nearest Marker	LOD	*R^2^*%	*A*
qBBR1	1	RM128–R01D144	RM128	10.1	21.5	4.89
qBBR3	3	R03D158–RM85	R03D158	6.2	12.3	3.73
qBBR5	5	RM7081–RM3616	RM6972	17.8	39.2	7.36

QTL, quantitative trait locus; BB, bacterial blight; LOD, log_10_ likelihood; *R^2^*, the proportion of variance explained by the QTL; A, additive effect at the QTL.

Because ASH1 has broad-spectrum resistance to different *Xoo* strains, P10 was selected as a representative to be used in QTL mapping, resulting in the detection of three major QTLs. However, it is possible that more QTLs for BB resistance would be identified if other *Xoo* strains were used to characterize the F_2_ population because of the race specificity of the resistance loci. The race specificity to different *Xoo* strains of the three detected QTLs also needs to be examined despite the possibility of their broad-spectrum resistance. For future work, we have developed a larger segregating population, and are performing further fine whole-genome wide QTL mapping and related studies including race specificity examination of different QTLs. The purpose is to confirm the BSA results and to provide a solid basis for the resistance gene cloning.

## 3. Discussion

Many wild species of rice possess genes for resistance to BB and four genes have been identified. The first of the genes, *Xa21*, was discovered in *O. longistaminata*, and encodes for a receptor kinase-like protein with a leucine-rich repeat domain that determines race-specific recognition [5,23]. Another BB resistance gene *Xa27* was cloned from *O. minuta* and its product is a nuclear localized type-III effector [9,24]. The other two genes, *Xa23* and *Xa29* were identified in *O. rufipogon* and *O. officinalis*, respectively; they have not been cloned but fine mapped in the relative chromosome regions [25,26]*.* These four genes have been introduced into cultivated rice through breeding programs and many resistant varieties have been developed for sustained improvement of rice productivity.

*O. meyeriana* is also an important wild relative of rice, and there have been many reports that it possesses high resistance to rice BB disease. Early in 1982 Peng *et al.* evaluated the resistance of *O. meyeriana*, *O. officinalis* and *O. rufipogon*, and found that *O. meyeriana* was highly resistant or nearly immune to BB disease [15]. Zhang *et al.* demonstrated that *O. meyeriana*’s resistance was ranked the highest among 871 accessions of 13 wild rice species [16]. Many subsequent studies have confirmed this high resistance of *O. meyeriana* [17,18,27]. In agreement with previous research, the results of this study also showed the trait of high BB resistance in this wild rice (Figure 1). These results suggest that *O. meyeriana* is a valuable genetic source that can be used for improving the BB resistance of cultivated rice. Additionally, *O. meyeriana* showed a wide resistance spectrum with strong resistance to all of the ten *Xoo* strains (Figure 1). This multiple resistance trait is particularly desirable in rice breeding for durable resistance to this disease. However, although the high resistance has been widely identified, no resistance gene(s) have yet been cloned in *O. meyeriana*, which has delayed the utilization of this important material in breeding programs.

At least 36 BB resistance genes have been identified in rice [1,2,3,4], and eight of them have been cloned by map-based cloning, including *Xa1*, *Xa3/Xa26*, *Xa5*, *Xa10*, *Xa13*, *Xa21*, *Xa25* and *Xa27* [3,4,5,6,7,8,9,10]. *Xa1* is located on chromosome 4 [6], *Xa3/Xa26* on chromosome 11 [28,29], *Xa5* on chromosome 5 [30], *Xa10* on chromosome 11 [4], *Xa13* on chromosome 8 [31], *Xa21* on chromosome 11 [32], *Xa25* on chromosome 12 [33], and *Xa27* on chromosome 6 [24]. *Xa5* and the resistance locus qBBR5 identified in the present study were both mapped to chromosome 5, but their mapping regions were different. *Xa5* was mapped in a 0.5 cM interval between the markers RS7 and RM611 on the front of the short arm of chromosome 5 [30], while qBBR5 was mapped in the interval between RM7081 and RM3616 on the long arm (Figure 6). Except *Xa5*, none of other seven genes shared the same chromosome as qBBR1, qBBR3 and qBBR5 that were identified in this study (Figure 6). Therefore, all three QTLs identified here are different from the loci of the eight *Xa* genes cloned in previous studies. In addition to the eight *Xa* genes mentioned above, more than 30 genes conferring BB resistance have been identified in different rice materials, but none of them have yet been cloned. These genes were mapped to different chromosomes from those on which qBBR1, qBBR3 and qBBR5 are located (1–4, Figure 6), except that *Xa29* shares the same chromosome as qBBR1 (26, Figure 6). Tan *et al.* mapped *Xa29* within a 1.3 cM interval flanked by the molecular markers C904 and R596 [26], a different region of chromosome 1 from the marker interval between RM128 and R01D144 to which qBBR1 was mapped (Figure 6), indicating that the loci of *Xa29* and qBBR1 are also different. Taken together, qBBR1, qBBR3 and qBBR5 are three novel resistance loci that are different from any known ones associated with resistance of rice to BB disease.

ASH1 is one of the hybrid progenies of asymmetric somatic hybridization between *O. meyeriana* and the cultivated rice Dalixiang [17]. Dalixiang was susceptible to BB disease, while ASH1 possessed the multiple resistance of the wild rice (Figure 1), indicating that the resistance gene(s) were transferred into ASH1 from the wild rice through somatic hybridization. Three novel QTLs were mapped in ASH1 in the present study and ASH1 alleles at any of the three QTLs contributed to the increased resistance (Figure 6; Table 1). Based on these results we suggest that the three QTLs identified in ASH1 were the ones transferred from *O. meyeriana* corresponding to the loci of the novel resistance genes in this wild rice. Our previous studies have revealed that the high resistance of *O. meyeriana* to rice BB disease results from multiple causes [34,35], which suggests that this wild rice may contain more than one gene conferring its strong resistance. This is consistent with the multiple resistance loci detected in ASH1 in this study. *O meyeriana* can hardly be crossed with cultivated rice by traditional hybridization, so little progress has been made on mapping and cloning its resistance genes. ASH1 inherits the high resistance from *O. meyeriana* and can be easily crossed with cultivated rice, and so it is an ideal intermediate material that can be used for cloning the resistance genes. The QTL mapping for BB resistance in ASH1 in the present work provides an important basis for further cloning the associated genes of *O. meyeriana*.

## 4. Experimental Section

### 4.1. Plant Materials

*O. meyeriana*, a wild species of rice with high BB resistance, was provided by Professor Xinhua Wei, China National Rice Research Institute (Hangzhou, China). *O. sativa* ssp. *japonica* (cv. Dalixiang) is a high-yielding, good-quality-grain cultivar grown in southern China, but it is susceptible to rice BB disease. ASH1 is derived from asymmetric somatic hybridization using *O. meyeriana* as the donor and Dalixiang as the recipient, and combines the high BB resistance of *O. meyeriana* with the good agronomic characters of Dalixiang [17]. *O. sativa* ssp. *indica* (cv. IR24) is an elite cultivar developed by International Rice Research Institute but highly susceptible to BB disease. Dalixiang, ASH1 and IR24 were introduced from the Zhejiang Academy of Agricultural Sciences collection. An F_2_ population including 101 individuals was developed from self-pollinations of the F_1_s of the cross between ASH1 and IR24. All the materials were sown in the seedling nursery and 25-day-old seedlings were transplanted into the experimental field in Zhejiang Academy of Agricultural Sciences, Hangzhou, China. In each experiment the plots were arranged in a randomized complete block design, and the spacing was 20 cm between plants within each row and 35 cm between rows. Field management followed normal agricultural practice.

### 4.2. Pathogen Inoculation and Resistance Identification

The *Xoo* strains used in this study included P2 (PXO86), P3 (PXO79), P4 (PXO71), P5 (PXO112), P6 (PXO99), P7 (PXO280), P8 (PXO145), P9 (PXO87), P10 (PXO124) and C4 (ZHE173) (Table 2). The inoculation was carried out using the leaf-clipping method as previously described [36,37]. Briefly, at the booting stage (approximately 40 days after transplanting), the fully expanded leaves were clipped about 1 cm from the leaf tip using a pair of scissors dipped in the inoculum. The bacterial inoculum was prepared from a 48 h culture on potato semisynthetic agar (PSA) [37] slants and its density was adjusted to 10^9^ CFU/mL. For resistance evaluation of the parents, *O. meyeriana*, Dalixiang, ASH1 and IR24 were inoculated with 10 *Xoo* strains. At least 16 leaves from four plants were inoculated with each strain. For resistance identification of the individuals in the F_2_ population, the plants were inoculated with *Xoo* strain P10, and at least 8 leaves were inoculated for each plant. Leaf length and lesion length were measured three weeks after inoculation. Additionally, electron microscopy was used to detect the sub-cellular location of *Xoo* pathogens in each plant.

**Table 2:** *Xoo* strains used in this study.

*Xoo* Strain	Origin
P2	Philippines
P3	Philippines
P4	Philippines
P5	Philippines
P6	Philippines
P7	Philippines
P8	Philippines
P9	Philippines
P10	Philippines
C4	China

### 4.3. Electron Microscopy

Ultrathin sections and electron microscopy were done as described by Hong *et al.* [38] with some minor modifications. Leaf segments (1 × 3 mm) were fixed with 2.5% (*v*/*v*) glutaraldehyde in 0.1 M phosphate buffer (pH 7.2) for 2 h at 4 °C. After post-fixation for 1 h at room temperature in phosphate buffer (pH 7.2) containing 1% (*m*/*v*) osmium tetroxide, the segments were dehydrated through an ethanol series, 15 min for each step, and finally dehydrated twice in 100% ethanol for 20 min. The samples were infiltrated in a mixture of acetone and Spurr resin, 1:1 (*v*/*v*) for 1 h and 1:3 (*v*/*v*) for 3 h at room temperature, and then in absolute Spurr resin overnight. The segments were then embedded in Spurr resin at 37 °C for 12 h followed by 78 °C for 8 h for polymerization. The polymerized blocks were cut into 70 nm ultra-thin sections with the ultramicrotome. Finally, the sections were stained with uranyl acetate and lead citrate for 15 min, and were then observed and photographed with an electron microscope (JEM-1200EX, JEOL, Tokyo, Japan).

### 4.4. BSA Selection of Linkage Markers

DNA was extracted from fresh young leaves of rice using a modified hexadecyltrimethylammonium bromide (CTAB) method described by Wu *et al.* [39]. Twenty resistant and 20 susceptible individuals were selected from the F_2_ population, respectively, and two DNA bulks were made by mixing equal amounts of DNA from these selected plants. A total of 698 SSR and STS markers evenly distributed on 12 chromosomes were pre-surveyed between the two parents for screening of polymorphism markers. The inter-parent polymorphic markers obtained were then surveyed in the two parents and two bulks for selecting the markers linked to BB resistance.

### 4.5. Genetic Map Construction and QTL Mapping

The genotypes of the linkage markers obtained were identified in the F_2_ population, and the genetic map was constructed by using MAPMAKER/EXP 3.0b [40]. The Kosambi mapping function was used to transform the recombination frequency to genetic distance (cM). The computer program Windows QTL cartographer 2.5 was used to perform QTL analysis in the F_2_ population as described previously [41]. The composite interval mapping (CIM) analysis was applied to map QTLs and estimate QTL effects. The QTL main effects were estimated using the maximum-likelihood estimation method. The logarithm of odds (LOD) threshold at the experiment-wise significance level of 0.05 was determined by using the permutation method [42]. LOD peaks for each significant QTL were used to position the QTL on the genetic map, and the additive effect (*A*) and the contribution of each QTL to the total variance (*R^2^*) were determined.

## 5. Conclusions

*O. meyeriana* is highly resistant to rice BB disease, but no resistance genes have yet been cloned in this wild rice, which has delayed the utilization of this important material in rice breeding programs. Previously, we introduced the BB resistance trait from *O. meyeriana* into a cultivated rice using asymmetric somatic hybridization. In this work, we analyzed the BB resistance of ASH1, one of the hybrid progenies, and found that ASH1 inherited the multiple resistance from *O. meyeriana*. An F_2_ population was constructed from the cross between ASH1 and a rice cultivar with high BB susceptibility. Based on BSA, 17 molecular markers that were linked to rice BB resistance were obtained using the individual plants with extremely high resistance and susceptibility selected from the F_2_ population. QTLs mapping was then performed based on the 17 linkage markers and the F_2_ segregating population, and finally three QTLs, qBBR1, qBBR3 and qBBR5 were identified on chromosomes 1, 3 and 5, respectively. All three QTLs were important gene loci having a strong effect on the BB resistance, and ASH1 alleles at any of the QTLs contributed to the increased resistance. These QTLs were three novel loci, differing from any known genes previously identified. The results will provide an important basis for further cloning of resistance genes in *O. meyeriana* and their utilization in rice resistance breeding.

## Supplementary Files

Supplementary File 1
