# Supplementary material for: Quantitative Trait Loci Mapping for Bacterial Blight Resistance in Rice Using Bulked Segregant Analysis
Source: Int J Mol Sci. 2014 Jul 3;15(7):11847–61. doi: 10.3390/ijms150711847 (PMC4139818; doi:10.3390/ijms150711847)
Supplement: Supplementary File 1 [file ijms-15-11847-s001.pdf]

Supplementary Information

Table S1. Molecular markers with polymorphism between the parents of ASH1 and IR24.

Marker	Chromosome	Position (Mb)	Type	Forward Primer (5'–3')	Reverse Primer (5'–3')
RM1282	1	0.5	SSR	AAGCATGACAGCTGCAAGAC	GGGGATGAAGGGTAATTTCG
RM6515	1	1.3	SSR	CTCGGCTAGTGACGATTTCTTGG	ACGTCGTGGTAGGCGACATAGC
RM5302	1	4.1	SSR	TATGGGTGACACATTGGGAC	TTGTGACGTTTGAGAGCTGG
RM1	1	4.6	SSR	GCGAAAACACAATGCAAAAA	GCGTTGGTTGGACCTGAC
RM522	1	5.2	SSR	ACCAGAGAAGCCCTCCTAGC	GTTCTGTGGTGGTCACGTTG
RM490	1	6.6	SSR	ATCTGCACACTGCAAACACC	AGCAAGCAGTGCTTTCAGAG
R01D56	1	10.6	STS	ATTCCTGGTTCTACATTACTTA	CGCCTCACTAGAATATCGGA
RM1287	1	10.8	SSR	CCATTTGCAGTATGAACCATGC	ATCATGCAATAGCCGGTAGAGG
RM3412	1	11.6	SSR	TGATGGATCTCTGAGGTGTAAAGAGC	TGCACTAATCTTTCTGCCACAGC
R01D73	1	16.2	STS	CTTCCTTGCTGGAGAGCTG	TTATGATGGTGCCTCCCATT
R01D95	1	23.2	STS	GTAAAGCATGCGTGCACACC	TTAATAAGAGGCAACGGAAC
RM1349	1	25.0	SSR	ATCCACCTGCTGATCAGCTC	CGAGAAGCTCAAGGTGAACC
RM5461	1	26.9	SSR	GTGGAGGAGCGAAGGGAACACG	CCTCCCATATAAACCGGCGAACC
R01D124	1	31.7	STS	ATAGTTCGCCATCGTCAT	ACACGCCATAGCAAGGAA
RM128	1	32.5	SSR	AGCTTGGGTGATTTCTTGGAAGCG	ACGACGAGGAGTCGCCGTGCAG
R01D144	1	38.5	STS	AATAGAATTACTGATGAAACCTTA	GCCCGTTACCGCTTATGT
RM1361	1	40.8	SSR	ATGCTTGCAGACAATCGATGC	CTCTCCGCCTAAACAACTTGTGC
RM5310	1	41.2	SSR	GGGACCAAGACCTTTCCAATGC	GCGGAAGCAGGAGAATCGTAGC
R01D182	1	42.9	STS	GTAATTGAATTTCACTGCTGCT	ACGTACGTGACGTGCTTATG
RM154	2	1.0	SSR	ACCCTCTCCGCCTCGCCTCCTC	CTCCTCCTCCTGCGACCGCTCC
R02D10	2	2.6	STS	GCACATCGGTAACGGTAGAGG	CGGTGGATAAAGACAAAGAGG
R02D23	2	4.6	STS	ACCAAGATCCTGGAGGCATT	TCCAACATGGCCACAGATTA
R02D37	2	6.4	STS	CCCAGTCTGCTGCCATCT	GAATGTATTTCAGTTCCAGTAAG
RM5699	2	9.0	SSR	TATTGGTATGTGTGGGATCG	CTGGTGTACCTATATGGATTGC
RM521	2	10.8	SSR	TTCCCTTATTCCTGCTCTCC	GGGATTTGCAGTGAGCTAGC
RM290	2	10.8	SSR	ACCCTTATTCCTGCTCTCCTC	GTGCTGTAGATGGAAGGGAG
R02D55	2	15.2	STS	GCAGCAAAGTGCGGAGTA	CAGGTGAATTGCCAATTT

Table S1. *Cont.*

Marker	Chromosome	Position (Mb)	Type	Forward Primer (5'–3')	Reverse Primer (5'–3')
R02D75	2	19.7	STS	CCACATCCTCTCATCTCTGTCA	CGACAGGTTCAGCTTCAGGT
RM3688	2	22.4	SSR	GTTGAATCAAGCTGTGCAGC	AGCTAGGCAAAGCATGCATG
RM6617	2	24.8	SSR	CTCCTCCTCCCACCTCTACTCC	TAATAGATGGCGATGGACGAAGG
RM6465	2	28.4	SSR	CCTAGGTTCCCATCGCAACTGACC	TTCCCGAGGAGGGAGACGAAGG
R02D129	2	30.3	STS	CCTGAAGGAAATGATAGCAATAG	GTTTTGTATGCTCTTCACTTGTC
RM240	2	31.5	SSR	CCTTAATGGGTAGTGTGCAC	TGTAACCATTCCTTCCATCC
RM425	2	32.3	SSR	ACCACAGCAGGTGGAACAGG	GCTAGCTAAGCCAACACCAACG
R02D144	2	33.5	STS	AGTCCACACGCCATCGTC	TCGCAGAACTCCAAACACAC
RM166	2	34.3	SSR	GGTCCTGGGTCAATAATTGGGTTACC	TTGCTGCATGATCCTAAACCGG
RM48	2	35.5	SSR	TGTCCCACTGCTTTCAAGC	CGAGAATGAGGGACAAATAACC
RM6349	3	0.8	SSR	ATGATGCCTCATGTCTCTGATCTCC	AGATGAACACGACCGATAGGATAGG
RM569	3	1.9	SSR	CTGCGTCAGATTTCTCCTCTTCG	ACATTCTCGCTTGCTCCTCTCG
RM6038	3	4.8	SSR	CCGTCGTGTCGTACTGGTAGTCC	TCCACAGCCTACCTAGCTTCTCC
RM1022	3	7.2	SSR	GTCTTTGATAGCGGCTTTGTCC	GGATGAGGGAGTAATGTCTCTTGG
RM232	3	9.7	SSR	CCGGTATCCTTCGATATTGC	CCGACTTTTCCTCCTGACG
RM5955	3	11.2	SSR	TCGCCGTAGGGCCAGTAGAAGC	CATCCACAACCTCCTGCAGTTCC
RM282	3	12.4	SSR	CTGTGTCGAAAGGCTGCAC	CAGTCCTGTGTTGCAGCAAG
RM6929	3	12.9	SSR	TTCTTTCGAGGGTACGTAGAGG	CTAGCTAGCCAGTAGCTGATCG
RM1164	3	14.7	SSR	TTTCTGGCGACGTGATTTGTCG	CAATTCGGAAGAGCAAACATGACC
R03D86	3	19.7	STS	AGGCTAAGTGAAGAAATAATAAG	CTCCGTATTCATTACTGGTTG
RM16	3	22.9	SSR	CGCTAGGGCAGCATCTAAA	AACACAGCAGGTACGCGC
RM6266	3	23.6	SSR	CACCTTCTTGAGAAGCTCCTTCG	GACATCGAGAGCGAGGACAGC
R03D110	3	26.3	STS	GCATTGAATTGTACTCTTATTATAT	ACGAATCAAAAGGAGACTAAAAT
R03D120	3	27.4	STS	TGGCACAAATCATTATGATC	GATTGCAATGCAGCATATAG
RM532	3	28.1	SSR	TCTATAATGTAGCCCCCCCC	TTTCAGGGGCTTCTACCAAC
R03D143	3	32.8	STS	AGGAGAAGCTCGCCATGAT	CATGAACTGCAACATCACCAG
R03D146	3	33.5	STS	ACACTGGCTACGGCAAAG	TTTGTTCGGGAATAATGATGC
RM143	3	34.1	SSR	GTCCCGAACCCTAGCCCGAGGG	AGAGGCCCTCCACATGGCGACC

Table S1. *Cont.*

Marker	Chromosome	Position (Mb)	Type	Forward Primer (5'–3')	Reverse Primer (5'–3')
R03D158	3	36.2	STS	CTGCAGGGACACCGAGAT	CCACAGCGACATCCTGTACC
R03D159	3	36.3	STS	CGACGGCAGATACGATACAC	CACCGCTGTTCAAAGTTTGTC
RM85	3	37.2	SSR	CCAAAGATGAAACCTGGATTG	GCACAAGGTGAGCAGTCC
RM7585	4	0.2	SSR	TGCAATCGCGTAGTTGGTAGAATAGG	CTCGACTACCTCGCCATCATCC
RM3216	4	1.1	SSR	AAGTCCGGTGACTCAGTAATCACG	GCTATATCCGGGCTCTTTCTTGC
RM261	4	6.5	SSR	CTACTTCTCCCCTTGTGTCG	TGTACCATCGCCAAATCTCC
R04D20	4	11.6	STS	AGTGCTCGGTTTTGTTTTC	GTCAGATATAATTGATGGATGTA
R04D35	4	18.1	STS	GCTTCTCCTGGTTGTATGC	AAAATAGGGAGGCAGATAGAC
R04D76	4	24.6	STS	CTTGAACCTGAGTGAGTGG	CGATGAAAATGATGTCTA
RM3288	4	27.5	SSR	CAATCTGGAGGCACTGTCACG	AGTGACAAGATGAAGCCAACAGC
R04D95	4	29.0	STS	TTTTGTGAAACTTGACCCTC	GCGTCCATGTCTTTATTGTG
RM3217	4	30.3	SSR	GACCTAACCAATCGGACGAACG	CACTAGTGCTCTGTCTCTGCTTACCC
RM348	4	32.9	SSR	CATGAAGCTGTGTTGCTGTTGC	CGCTACTAATAGCAGAGAGACCATCG
R04D123	4	34.0	STS	GAAGATTTTGCGTGTTGCTG	TCCGATAGCCCATCTGTACC
RM122	5	0.3	SSR	GAGTCGATGTAATGTCATCAGTGC	GAAGGAGGTATCGCTTTGTTGGAC
RM413	5	2.2	SSR	CCAATCTTGTCTTCCGGATCTTGC	AGATAGCCATGGGCGATTCTTGG
R05D22	5	2.6	STS	GGGGTCGATTTCAAGTGGTA	GGAAGATGAAGACGGTGGTG
RM5874	5	3.5	SSR	GAAATCCCATTTCGTTGCTGATGG	CACTCAGCATCATCGCCAGAGC
R05D41	5	5.9	STS	GAGAAAGAGTGGAAGGAG	AGTATCGTCAGGAGGGTC
RM5994	5	6.8	SSR	ATCAGGCACTGCAGCATGTCG	TTGAACAACTCGTCACCCTCATCG
RM289	5	7.8	SSR	TTCCATGGCACACAAGCC	CTGTGCACGAACTTCCAAAG
R05D55	5	13.9	STS	CTCGCTGTTTACTGACTGG	TTTGATGTACTGCCTGCTCT
RM6645	5	14.9	SSR	CTCCGGGATGCCATAGTTTCG	AAGCTTCCTCTCGATCGTCTTCG
RM430	5	18.6	SSR	GTCCCTGATCAGAAACGAGATGG	TAGGGTTGGAAGAATGCAAGACC
R05D87	5	21.5	STS	CTCAATTTCACCCATCCC	CGCTCCGTCTCCAACCTC
RM5642	5	22.1	SSR	AAAAACCGGCTAATCCCTCC	TTCGATGGGATTGATCGC
RM3870	5	22.8	SSR	GGAGTAGATGTAAAGCCAAAGGATGC	CATGTCTGAGTATGACGGAGTATTGC
RM3476	5	24.0	SSR	TGATAGTTGACAATGCAGGAGAGG	TCGATCCGGAAGTTATTTCTGC

Table S1. *Cont.*

Marker	Chromosome	Position (Mb)	Type	Forward Primer (5'–3')	Reverse Primer (5'–3')
RM7081	5	24.7	SSR	CTTCCCGCACTACACTGCACTCC	CTGCAACTTGCTCATGGAGTTGG
RM3348	5	25.2	SSR	CTTCTCGGTTCATCCAAAGAGC	GTGGAAGCTATGGGTAGCTCACG
RM6972	5	25.5	SSR	CATGGTGCTCCTACTGGTTGTACC	CCCATCCATAATCACAACTCAGC
RM3321	5	25.9	SSR	CTATAAATAGGGCCAGGTGGTAGGC	CTGCCTAGCCATAGCCAAACG
RM3616	5	26.4	SSR	ACACCAGCGACGATCGATTCC	CTGCGTGAGACGGTTGACTGACC
RM6360	5	28.3	SSR	ACGTGGAATCCAAATTGACAGC	TTCGCTGCACTGTTTACTCTTGG
RM334	5	28.6	SSR	GTTCAGTGTTCAGTGCCACC	GACTTTGATCTTTGGTGGACG
RM170	6	1.3	SSR	TCGCGCTTCTTCCTCGTCGACG	CCCGCTTGCAGAGGAAGCAGCC
RM510	6	2.8	SSR	AACCGGATTAGTTTCTCGCC	TGAGGACGACGAGCAGATTC
RM3805	6	2.9	SSR	ACACCACCATCAACGTACCAACC	AAGTCGAGAGGAAGAAGCCAAGG
RM585	6	3.1	SSR	CAGTCTTGCTCCGTTTGTTG	CTGTGACTGACTTGGTCATAGG
RM6773	6	4.4	SSR	GCTGCTCCACCTTCACCTTCC	CGATGGTGTGTTGTTTGGTTGC
RM50	6	6.3	SSR	ACTGTACCGGTCGAAGACG	AAATTCCACGTCAGCCTCC
RM539	6	8.1	SSR	GAGCGTCCTTGTTAAAACCG	AGTAGGGTATCACGCATCCG
R06D51	6	8.1	STS	GGCAACCGACCATCACTTAG	CCCCACTGGTCAAATGAGTT
RM3183	6	12.3	SSR	GTGGTGCTAGTATGGACGAGAGG	CGGTTGGTAGACTGTAAACAAAGTGC
RM1340	6	23.0	SSR	ATCGATCTCCACCACTTCCTTCC	CCCTACTCCCAGTAACCCAAATAGG
RM7434	6	23.6	SSR	AGGCTTCTTGGAATGGAACTGC	GGGAATATACGTGGATGTGAGAGG
RM162	6	24.0	SSR	GCCAGCAAAACCAGGGATCCGG	CAAGGTCTTGTGCGGCTTGCGG
RM6395	6	25.6	SSR	GGCTTCGGCTTCTGAACTAGC	CGACTAAGCAGCAGTAACAATCTCG
RM1370	6	28.1	SSR	AAACGAGAACCAACCGACAC	GGAGGGAGGAATGGGTACAC
RM340	6	28.2	SSR	GGTAAATGGACAATCCTATGGC	GACAAATATAAGGGCAGTGTGC
RM439	6	29.1	SSR	CTGGGTCTAATCTCGTCCTAAATTGC	CGCCTCTCATAACAGTCCACTCC
RM5814	6	29.4	SSR	GATCTCCACCACCTCCATCTCC	CCTACATCAAGGCTCGCTACTGC
RM5344	7	1.9	SSR	ATGACCTTCCTCGCAAGAGTGC	ACACAAGGCTCTCAACCAAGACG
RM427	7	2.7	SSR	TTGAGCTGATGAGAGTTGGTTGC	CTGTCACTAGCTCTGCCCTGACC
R07D25	7	3.1	STS	CCTGGGACCTTGTACCCTCT	TGACACCATCAGAAAACTGCT
R07D44	7	6.7	STS	ACCTTCCCTCCCCTTTTGAT	AACTTGGTCTTCCTGTTTTATTG

Table S1. *Cont.*

Marker	Chromosome	Position (Mb)	Type	Forward Primer (5'–3')	Reverse Primer (5'–3')
RM3755	7	14.7	SSR	TGTGGACAACCTCAACTGAAAGC	CATAATCACCAACATCGGAGAAGC
R07D68	7	19.4	STS	ATGTCGCCTACGAGTTTTC	TTCATGTGACCATTTGTGC
R07D82	7	23.6	STS	CAGCCCTAAATCTAAATACCC	ACGTTGAGACAGGCGAGC
R07D99	7	26.4	STS	CCGGTGACTTCTTCATGTCC	GCACACTCATCGGTGCATAC
R07D100	7	26.7	STS	TGTTTTTGTTGCTCCCAGTG	GGGAGAACCTTCCCACTGAT
RM172	7	29.5	SSR	TGCAGCTGCGCCACAGCCATAG	CAACCACGACACCGCCGTGTTG
RM3710	8	0.4	SSR	AGCAGCAGCCGCTTCTTGTCG	CGATTGTTTCCTCCGCCATTCC
RM6393	8	0.6	SSR	TCTGGATGTAGTCGATGCTGAGG	AGAAGAGAGCAGCGTGACATGG
RM152	8	0.6	SSR	GAAACCACCACACCTCACCG	CCGTAGACCTTCTTGAAGTAG
R08D17	8	2.1	STS	TCTGGACTGGAAATAGCATGG	GTGTGAAAGCCCATCTGTCA
RM1376	8	3.2	SSR	ATGCATGTGTGATGACTGACAGG	GGTACTCTTGCCAAATGGTCTCC
RM3572	8	3.9	SSR	CCATTTGGTAGGTCCATCTTACCC	CTCCCAAGTGAAGTGCTGTCTGG
RM3181	8	7.5	SSR	TTTAGCGAAGCAAACCCTCACC	CGAGCCAAGAAGAATGAATCAGC
RM8271	8	7.6	SSR	AGCAGCTCCGATTGTGTTAGCC	AATGGCGTCTGTGGTACTTTGC
R08D50	8	8.8	STS	GGAGGAGGAGGAGAGATGGT	GCAGCAGAACATGAGTTTGG
R08D54	8	13.4	STS	CCTATTCACTCTACCGACAT	GTTTAGTTCCCATTGCTTT
RM223	8	20.6	SSR	GAGTGAGCTTGGGCTGAAAC	GAAGGCAAGTCTTGGCACTG
R08D77	8	20.7	STS	CGAAAGAGGAGAGGGGTAGT	CGAAAACGAGAAACAAATA
RM284	8	21.1	SSR	ACTGCATGATCCTCCTCAGATCC	CCCTCTGATCTCTGATACTCCATCC
RM3262	8	22.4	SSR	CTGGAGATGCAGATCCTCAACC	TAGTACAACATGGGAGCCTGTCG
RM6976	8	23.6	SSR	CTGCAACCTGCACGAGTACACC	GTCCCATTGGATAGAATCCCAGAGC
RM230	8	25.8	SSR	GCCAGACCGTGGATGTTC	CACCGCAGTCACTTTTCAAG
RM7400	8	27.9	SSR	TTTGATTTGTGCAGGGATACGC	CTGCAGCAGAAACACGAAGAGG
RM264	8	27.9	SSR	GTTGCGTCCTACTGCTACTTC	GATCCGTGTCGATGATTAGC
RM316	9	1.0	SSR	CTAGTTGGGCATACGATGGC	ACGCTTATATGTTACGTCAAC
R09D28	9	9.4	STS	ACTGCTTTGATGGCTTGTG	CTCCCCAAACTGAATCC
RM105	9	12.5	SSR	GTCGTCGACCCATCGGAGCCAC	TGGTCGAGGTGGGGATCGGGTC
R09D51	9	14.7	STS	CTCACCTACCTAAAACCCAAC	CCACCCAAATCTGATACTG

Table S1. *Cont.*

Marker	Chromosome	Position (Mb)	Type	Forward Primer (5'–3')	Reverse Primer (5'–3')
RM3700	9	15.4	SSR	CCTTTGCCGCCTTCTCTTGG	ACGAGTTCCCGGTTAACCTTACG
RM257	9	17.7	SSR	CCGTGCAACTTAAATCCAAACAGG	GGAATCCTATATGAGCCAGTGATGG
R09D75	9	19.2	STS	CTATAAGACCAAAACGAAAACT	GAAAACCATTGTGTCACTGTA
RM107	9	19.8	SSR	TCTTACTGCGTCCTCTGGGTTCC	ATTCTTGCGGCGATTCATCTTCC
RM215	9	20.9	SSR	GAGCAGCAAGAGCAGCAGAGG	CATGCTCGACTTCAGAAGCTTGG
R09D92	9	22.5	STS	GACAGAGAAACTGGGCGTTC	CTGTTAAACCCCCACAAAGC
RM205	9	22.7	SSR	CTGGTTCTGTATGGGAGCAG	CTGGCCCTTCACGTTTCAGTG
RM6370	10	0.3	SSR	TTGACAAGCCACACACACAG	GTCCTCCCTTGGTTCTTTCC
R10D16	10	8.4	STS	AATGCCACATCTTCCTTCAAA	CAGGACAGAGGAGAGGGAGA
RM239	10	9.7	SSR	TACAAAATGCTGGGTACCCC	ACATATGGGACCCACCTGTC
R10D38	10	16.0	STS	TAAGACCTTTGCCTGCTGGT	GAGCAGATTTTTCCCACTGC
R10D45	10	17.4	STS	CAAGCCTGGATCACCATCTC	CTCCACAGCGGTAAGTGTTG
R10D57	10	19.4	STS	GTCCCTAGGCCATCTCTTG	GCGAATAGGGGTGGACAG
R10D61	10	20.3	STS	TGGGGAACTGTTGAAGATGA	ATCGTGGGCAAAGCAATACT
RM1374	10	21.6	SSR	TAGATATGTTGGGCCGGAAG	AGATCGATGCCGTTTCAGAC
RM591	10	22.5	SSR	CTAGCTAGCTGGCACCAGTG	TGGAGTCCGTGTTGTAGTCG
RM6824	10	23.1	SSR	GAGAGAACCTGGTGGTGGAG	AGTGGTAGAAGATCCGAGATCG
RM6395	10	25.6	SSR	GGCTTCGGCTTCTGAACTAGC	CGACTAAGCAGCAGTAACAATCTCG
RM7557	11	2.3	SSR	CCTCCAGGTGAAGTGCCTTTGC	CTTCATCTTCCCTGCTGCCTTTGC
RM1124	11	3.8	SSR	CTAGGGATCGGTAGACCCAATCG	TGGTGATGGCACTTTAGACAGAAGG
R11D24	11	4.3	STS	TTCGGCATCTACCATGACTG	CTTGATCTCGTCCACCGTCT
RM120	11	5.7	SSR	CACACAAGCCCTGTCTCACGACC	CGCTGCGTCATGAGTATGTA
RM3701	11	8.0	SSR	GAAAGAGGAGGAAGAGCTAGAGG	CCATATGTACGGAGTGTGTTTACC
R11D45	11	8.1	STS	AGTTTTGACTGATAGCCGATTG	GAGAAGAAAGAGGAGGAAGAGC
RM3428	11	13.4	SSR	GCCATTGACACCAAATGATCACC	GGCATATAAGGTCCATGGTGAATTGG
RM6272	11	16.4	SSR	ATCTACTCCGCCACCACCACAGC	CTCTACCTCTCCCTCTCGCCTTCC
RM287	11	16.6	SSR	TTCCCTGTTAAGAGAGAAATC	GTGTATTTGGTGAAAGCAAC
RM5349	11	19.0	SSR	CATCCAAATGTTGCGGATTACC	TTCAATAGCCCAGAGAACCAAGC

Table S1. *Cont.*

Marker	Chromosome	Position (Mb)	Type	Forward Primer (5'–3')	Reverse Primer (5'–3')
R11D80	11	19.9	STS	AGGAGATACAGGGCCAGGTT	CCCTTCAGGATCAAATGGAG
RM254	11	24.2	SSR	AGCCCCGAATAAATCCACCT	CTGGAGGAGCATTTGGTAGC
RM7170	11	24.4	SSR	GCGACTTGAGAGCGTTTGTAGG	AGCCAACTGTAGCACGAACTGC
R11D100	11	24.8	STS	AAGAAAAATATCTATTGAGGAGTG	GGAGGACCATAAATGACGG
R11D110	11	26.5	STS	GCGGAAAAGCAAGAGGAAT	GCCCATGAGAGAAGTAAAGGAA
RM2064	11	26.6	SSR	TTGATGTTGTGGGCTTCTCTACC	TCCAATCACGCGTATTACATGC
RM4A	12	0.9	SSR	TTGACGAGGTCAGCACTGAC	AGGGTGTATCCGACTCATCG
RM8216	12	1.4	SSR	AGTTGATTATCCATCATGTGC	TGTAGGCCCTATGAATCTCC
RM6296	12	3.2	SSR	TTAAGCCCACGTTTCTCTTGTCC	CTCGCTAGGGTTAGGGTTTCAGG
RM7619	12	4.8	SSR	TCTTGGTATGTATTGGCAGCGAAAGC	AGGATGTGAATGAAGGCGAATGG
RM7003	12	6.8	SSR	CTCTAGCTCTCTCATGGATGG	AATCATAGGGCAGACATACAGC
R12D51	12	11.4	STS	CCTAACAGCGGCATCTCCT	AGTGCATTCACCCAAAAATTG
RM277	12	18.3	SSR	CGGTCAAATCATCACCTGAC	CAAGGCTTGCAAGGGAAG
RM1246	12	19.2	SSR	GGCTCACCTCGTTCTCGATCC	CATAAATAAATAGGGCGCCACACC
R12D70	12	19.8	STS	TTGATGATAGTATTTGCTGATG	AGATAGTGTCGGCGGTGG
RM6947	12	24.0	SSR	GGCTGCTGGTATGTATTCAAGTGC	GCAAGTGAGAAACAGAAGTGATCG
R12D103	12	26.0	STS	CCGCCGAGAAGAAACAAAG	CCCAAGAACAGGATTACA
RM7558	12	27.0	SSR	CAGTAGCAGGCTCCCTTTTG	ATCAGGAACACCAGAGACGG
RM1227	12	27.4	SSR	CATCGACATGTGGACCACTCC	GCCTGAGACAAGTCCATGGTAGC

Table S2. Linkage markers to BB resistance

Marker	Chromosome	Position (Mb)	Type	Forward Primer (5'–3')	Reverse Primer (5'–3')
R01D124	1	31.7	STS	ATAGTTCGCCATCGTCAT	ACACGCCATAGCAAGGAA
RM128	1	32.5	SSR	AGCTTGGGTGATTTCTTGGAAGCG	ACGACGAGGAGTCGCCGTGCAG
R01D144	1	38.5	STS	AATAGAATTACTGATGAAACCTTA	GCCCGTTACCGCTTATGT
RM1361	1	40.8	SSR	ATGCTTGCAGACAATCGATGC	CTCTCCGCCTAAACAACTTGTGC
R03D143	3	32.8	STS	AGGAGAAGCTCGCCATGAT	CATGAACTGCAACATCACCAG
R03D146	3	33.5	STS	ACACTGGCTACGGCAAAG	TTTGTTCGGGAATAATGATGC
RM143	3	34.1	SSR	GTCCCGAACCCTAGCCCGAGGG	AGAGGCCCTCCACATGGCGACC
R03D158	3	36.2	STS	CTGCAGGGACACCGAGAT	CCACAGCGACATCCTGTACC
R03D159	3	36.3	STS	CGACGGCAGATACGATACAC	CACCGCTGTTCAAAGTTTGTC
RM85	3	37.2	SSR	CCAAAGATGAAACCTGGATTG	GCACAAGGTGAGCAGTCC
RM3476	5	24.0	SSR	TGATAGTTGACAATGCAGGAGAGG	TCGATCCGGAAGTTATTTCTGC
RM7081	5	24.7	SSR	CTTCCCGCACTACACTGCACTCC	CTGCAACTTGCTCATGGAGTTGG
RM3348	5	25.2	SSR	CTTCTCGGTTCATCCAAAGAGC	GTGGAAGCTATGGGTAGCTCACG
RM6972	5	25.5	SSR	CATGGTGCTCCTACTGGTTGTACC	CCCATCCATAATCACAACTCAGC
RM3321	5	25.9	SSR	CTATAAATAGGGCCAGGTGGTAGGC	CTGCCTAGCCATAGCCAAACG
RM3616	5	26.4	SSR	ACACCAGCGACGATCGATTCC	CTGCGTGAGACGGTTGACTGACC
RM6360	5	28.3	SSR	ACGTGGAATCCAAATTGACAGC	TTCGCTGCACTGTTTACTCTTGG
